# Gonadal Transcriptome Analysis in Sterile Double Haploid Japanese Flounder

**DOI:** 10.1371/journal.pone.0143204

**Published:** 2015-11-18

**Authors:** Xiaoyan Zhang, Jilun Hou, Guixing Wang, Hongbo Jiang, Yufen Wang, Zhaohui Sun, Xiufeng Jiang, Qinghai Yu, Haijin Liu

**Affiliations:** 1 Beidaihe Central Experiment Station, Chinese Academy of Fishery Sciences, Qinhuangdao, China; 2 College of Animal Science and Veterinary Medicine, Shenyang Agricultural University, Shenyang, China; 3 Centre for Applied Aquatic Genomics, Chinese Academy of Fishery Sciences, Beijing, China; Laboratoire de Biologie du Développement de Villefranche-sur-Mer, FRANCE

## Abstract

Sterility is a serious problem that can affect all bionts. In teleosts, double haploids (DHs) induced by mitogynogenesis are often sterile. This sterility severely restricts the further application of DHs for production of clones, genetic analysis, and breeding. However, sterile DH individuals are good source materials for investigation of the molecular mechanisms of gonad development, especially for studies into the role of genes that are indispensable for fish reproduction. Here, we used the Illumina sequencing platform to analyze the transcriptome of sterile female DH Japanese flounder in order to identify major genes that cause sterility and to provide a molecular basis for an intensive study of gonadal development in teleosts. Through sequencing, assembly, and annotation, we obtained 52,474 contigs and found that 60.7% of these shared homologies with existing sequences. A total of 1225 differentially expressed unigenes were found, including 492 upregulated and 733 downregulated genes. Gene Ontology and KEGG analyses showed that genes showing significant upregulation, such as *CYP11A1*, *CYP11B2*, *CYP17*, *CYP21*, *HSD3β*, *bcl2l1*, and *PRLR*, principally correlated with sterol metabolic process, steroid biosynthetic process, and the Jak-stat signaling pathway. The significantly downregulated genes were primarily associated with immune response, antigen processing and presentation, cytokine–cytokine receptor interaction, and protein digestion and absorption. Using a co-expression network analysis, we conducted a comprehensive comparison of gene expression in the gonads of fertile and sterile female DH Japanese flounder. Identification of genes showing significantly different expression will provide further insights into DH reproductive dysfunction and oocyte maturation processes in teleosts.

## Introduction

Double haploid (DH) fish are created by the process of mitogynogenesis. In mitogynogenesis, cell division is activated in haploid oocytes using irradiated sperm and diploidy is restored by temperature shock or hydrostatic pressure, which block the first cleavage to produce a diploid zygote. Offspring produced by mitogynogenesis are 100% homozygous, because a single set of chromosomes is duplicated [1]. Double haploidy is an advantageous situation for genetic mapping and genome sequencing studies [2]. As a rule, the effects of extremely high homozygosity are first noticed in fertility-related traits, especially in females [3]. In tilapia, only 10 of 77 gynogenetic DH females produced viable eggs [4]. In androgenetic common carp, the fertility rate was even lower with only 4 of 48 presumed females producing viable eggs [5]. The same fertility problem also occurs in Japanese flounder DH fish in which a few are fertile and the remainder are sterile and show varying levels of dysgenesis [6]. However, the sterile DH individuals do provide a good source material for research into the molecular mechanisms of gonad development, especially for identifying genes that are indispensable for fish reproduction. Reproductive processes in fish can be modified by environmental factors such as nutrition [7], temperature [8], and endocrine disruptors [9]. The majority of studies to date on female teleosts have investigated the effect of these factors on circulating sex hormone levels or on reproductive success in terms of spawning performance. Consequently, there are major gaps in our understanding of the molecular and cellular mechanisms of infertility.

RNA-seq has been used for quantitative gene expression analyses of biological processes in selected tissues or cells of a range of species [3, 10]. This method can be used to study genome-wide differences in gene expression through new generation high-throughput sequencing and information analysis platforms.

In the present study, we used RNA-seq to analyze the differential expression of genes in gonads of fertile and sterile female DH Japanese flounder. For some differentially expressed genes, real-time PCR was used to validate the differences. Our aim was to screen for the major genes that cause sterility in the DH flounder, and thus provide a molecular basis for an intensive study of oocyte maturation processes in teleosts.

## Results

### RNA-seq of sterile and fertile DH Japanese flounder

Two RNA-seq libraries were constructed from sterile and fertile fish using the Illumina Hi-Seq 2000 Genome Analyzer platform. This generated 5.1–7.2 billion clean reads sufficient for analysis of gene expression (Tables 1–3). These reads were then used in a blast analysis of target species with a cutoff of 1e-5. We identified 31,831 unigenes with homology to zebrafish transcripts, 30,147 unigenes with homology to mouse transcripts, and 30,264 unigenes with homology to human transcripts, accounting for 60.6%, 57.5%, and 57.7% of the total unigenes, respectively (Table 4). The gene ontology (GO) annotation process uses a dynamically structured control vocabulary that can be applied to describe gene function; the genes are first classified into three major categories, namely Biological Process, Molecular Function, and Cellular Component, and then into various sub-categories. Pathway Annotation is used to determine gene interactions in different metabolic pathways. We identified 29,416 unigenes assigned to biological process, 29,475 unigenes to molecular function, 29,464 unigenes to cellular component; 11,505 unigenes were assigned for further study by Pathway Annotation. Differential gene expression analysis in the control (fertile) group and case (sterile) group was performed using the DEseq Algorithm; 1225 differentially expressed unigenes were identified, of which 492 showed upregulation and 733 showed downregulation (see S1 File).

**Table 1:** Raw data and clean data statistics.

Sample name	Before filter (reads)	After filter (reads)	Base filter %
**F1**	5372771647	5198504943	96.77%
**F2**	6049180053	5866858828	96.99%
**F3**	5952567280	5765483482	96.86%
**S1**	5198681376	5044969881	97.04%
**S2**	7459885244	7191224734	96.40%
**S3**	7037492168	6821752968	96.93%

*Note: F1, F2, F3 and S1, S2, and S3 represent 3 fertile DH gonads and 3 sterile DH gonads, respectively; the same code is used in other Tables.

**Table 2:** Result of the Trinity transcripts assembly.

Sample name	F1	F2	F3	S1	S2	S3
**Raw reads**	53728010	60492160	59526022	51987136	74599184	70375224
**Clean reads**	52205350	58855834	57841646	50585486	72116290	68417302
**Number of contigs**	36499	37287	37645	36953	38528	39647
**Number of characters**	49304573	50626218	51400958	48397183	53368664	51946657
**Average contig length (bp)**	1350.85	1357.74	1365.41	1309.70	1385.19	1310.23
**Median contig length (bp)**	770	782	779	737	790	734
**Contig N50 length (bp)**	2493	2498	2515	2394	2544	2414
**Reads mapping to all unigenes (%)**	99.30	99.13	99.44	99.37	99.59	99.28

**Table 3:** Result of CAP3 clustering.

Sample name	All unigenes
**Number of contigs**	52474
**Number of characters**	75641345
**Maximum contig length (bp)**	17137
**Minimum contig length (bp)**	201
**Median contig length (bp)**	706
**Contig N50 length (bp)**	2895

**Table 4:** Unigene annotation and Blast result.

Unigene blast	Annotated gene	Unigene annotation	Annotated gene
**All unigenes**	52474	All unigenes	52474
**Blast to zebrafish**	31831	GO-Biological Process (BP)	29416
**Blast to mouse**	30147	GO-Molecular Function (MF)	29475
**Blast to human**	30264	GO-Cellular Component (CC)	29464
		Pathway	11505

### Functional enrichment analysis

**Fig 1:**
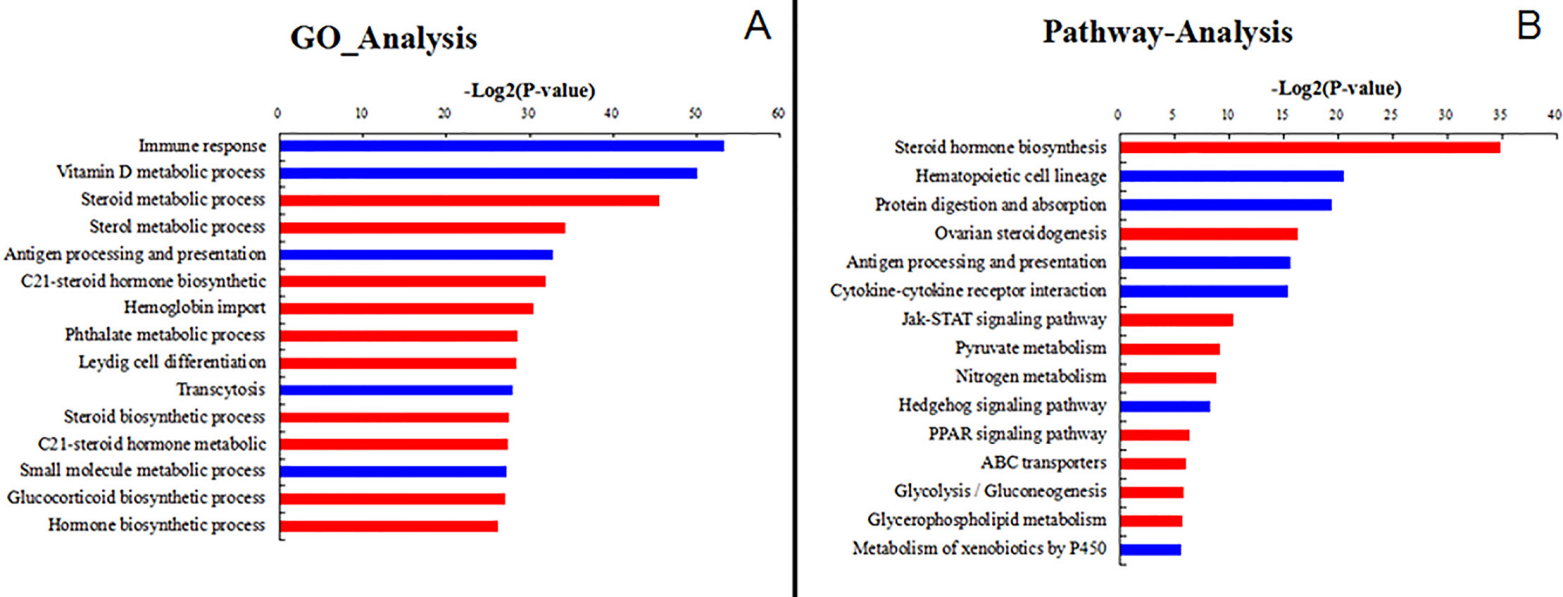
Gene ontology analysis and pathway analysis of all differentially expressed unigenes. (A) Gene ontology analysis of differentially expressed unigenes. The blue bar indicates up-regulated genes in sterile gonads compared with fertile gonads; the red bar indicates down-regulated genes in sterile gonads compared with sterile gonads. (B) Pathway analysis of all differentially expressed unigenes. The blue bar indicates up-regulated genes in sterile gonads compared with fertile gonads; the red bar indicates down-regulated genes in sterile gonads compared with sterile gonads.

Based on the results of the unigene annotation, a GO analysis of expressed unigenes was applied and significant *p*-values were confirmed by Fisher’s exact tests. Of 1225 differentially expressed unigenes, 573 unigenes were annotated to 351 significant biological process go(*p* < 0.01): 175 were enriched by over-expressed genes in sterile gonads, 278 were enriched by under-expressed genes in sterile gonads. Among these biological processes, the genes highly expressed in the sterile gonads were mainly associated with immune response, vitamin D metabolic process, transcytosis, and small molecular metabolic process; the highest expressed genes in the fertile gonads were closely related to sterol metabolic process, steroid metabolic process, and C21-steroid hormone biosynthetic process (Fig 1A). *CYP11A1*, *CYP11B2*, *CYP17A1*, *CYP21A1*, *HSD3β*, and *PRLR* were the six genes showing the highest expression in fertile compared to sterile gonads (see S2 File).

A pathway analysis using the KEGG database was used to identify the significant pathways involving differentially expressed gene sets. Out of 1225 differentially expressed unigenes, 301 were successfully annotated. The significance level was calculated by Fisher’s exact test. In total, 46 pathway categories were found to be significantly enriched, including 26 pathways significantly enriched by under-expressed genes in the sterile gonads and 36 by over-expressed genes. Steroid hormone biosynthesis was the most significant pathway (Fig 1B) with six differentially expressed genes (*CYP11A1*, *CYP11B2*, *CYP17A1*, *CYP21A1*, *HSD3B6*, and *AKR1D1*) that showed lower expression in sterile gonads. The pathways showing greatest downregulation were involved in immune response, protein digestion and absorption, and cytokine–cytokine receptor interaction. The genes *CTSS*, *CD74*, *B2M*, *C1QB*, *C1QC*, *SLC3A2*, *DPP4*, *CPA1*, *CTRB1*, *TGFB3*, *CSF3R*, and *TNFSF10* were involved in these pathways (see S3 File).

### Differential expression: keyword analysis

**Fig 2:**
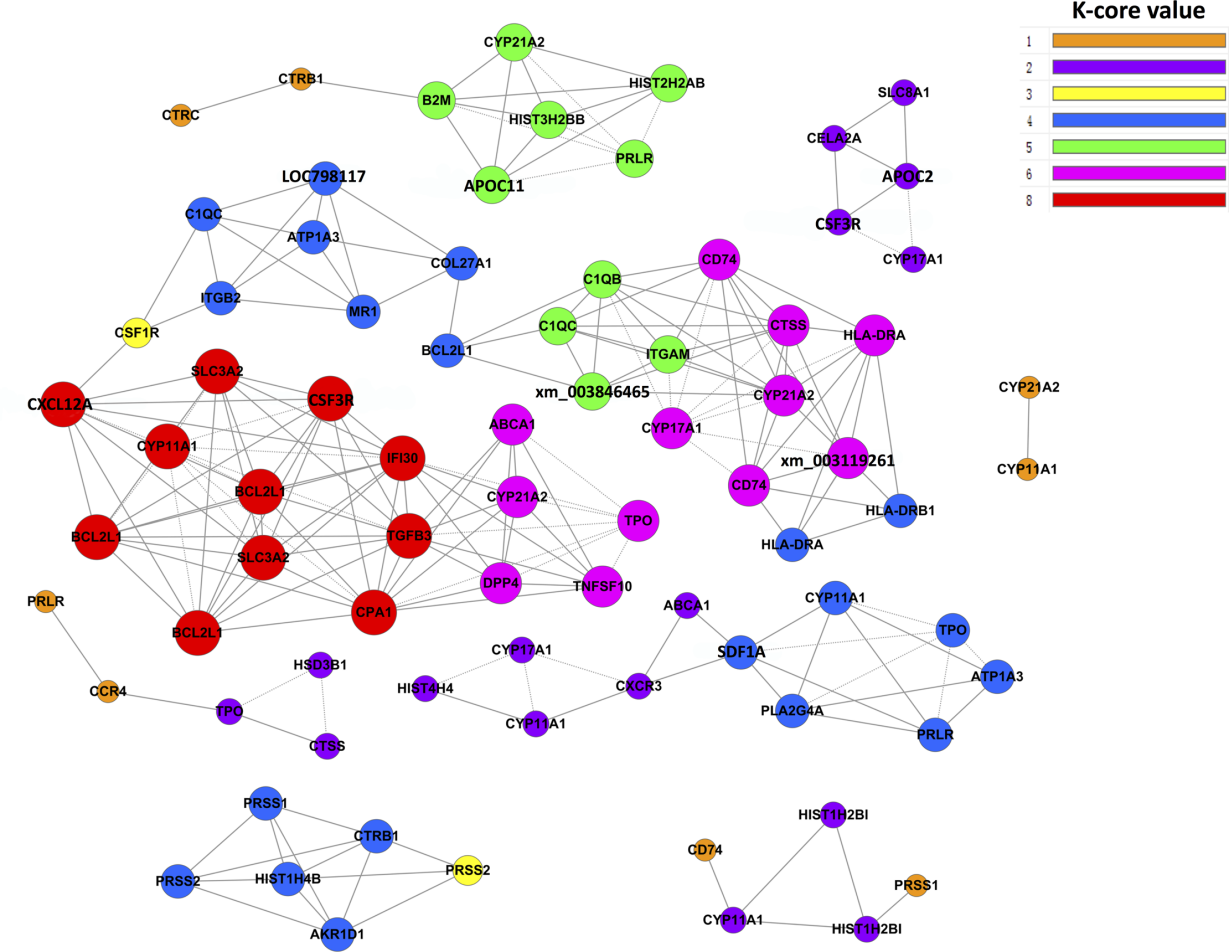
Co-expression network of differentially expressed genes in sterile gonads. Genes from pathways showing high expression differences were analyzed and identified using a gene co-expression network with a k-core algorithm. Cycle nodes represent genes, and the size of the node represents the power of the interrelationships among nodes; the edges between two nodes represent interactions between genes, and the greater the number of edges associated with a gene, the more it is connected to other genes and the more central is its role within the network.

Gene co-expression networks were constructed to identify pivotal gene or genes. In the network, cycle nodes represent genes, and edges between two nodes represent interactions between genes, which are quantified by degrees. Degrees within the network describe the number of single genes that regulate other genes and represent the size of the cycle node; the higher the degree, the more centrally the gene occurs within the network [11]. The network was constructed using 72 differentially expressed genes in sterile gonads (Fig 2). These genes were attributed to the pathways steroid hormone biosynthesis, *Staphylococcus aureus* infection, hematopoietic cell lineage, protein digestion and absorption, antigen processing and presentation, cytokine–cytokine receptor interaction, proximal tubule bicarbonate reclamation, and Jak-stat signaling. Three of these pathways were upregulated and five were downregulated. Core regulatory genes involved in the eight pathways were determined using k-core differences between the sterile and fertile groups. As shown in Table 5, *TGFB3* and *SLC3A2* had the biggest k-core differences, followed by *CYP11A1* and *DPP4*. Among these four genes, *CYP11A1* was upregulated and more highly expressed in the fertile group and at a lower level in the sterile group; it directly regulates 10 neighboring genes that interact according to their degrees. The other three genes are related to protein digestion and absorption and cytokine–cytokine interaction, and showed downregulated expression. Other upregulated genes with large k-core differences were related to steroid hormone biosynthesis and the Jak-stat signaling pathway, including *CYP21A1*, *CYP17A1*, *HSD3B6*, *CYP11B2*, and *PRLR*.

**Table 5:** Eighteen genes identified by gene co-expression network with k-core algorithm.

Gene symbol	Fertile		Sterile		Dif-k-core	Expression in sterile gonads relative to fertile gonads
	Degree	k-core	Degree	k-core		
***TGFB3***	1	1	14	8	-7	up
***SLC3A2***	1	1	9	8	-7	up
***CYP11A1***	3	2	10	8	-6	down
***DPP4***	0	0	7	6	-6	up
***CPA1***	3	3	13	8	-5	up
***CTSS***	2	1	10	6	-5	up
***BCL2L1***	3	3	10	8	-5	down
***CYP21A***	1	1	7	6	-5	down
***ABCA1***	1	1	6	6	-5	up
***CXCR3***	10	8	15	9	-5	up
***APOC1I***	1	1	5	5	-4	up
***CSF3R***	4	4	10	8	-4	up
***CYP17A1***	3	3	8	6	-3	down
***TNFSF10***	3	3	7	6	-3	up
***HSD3β***	5	4	2	2	2	down
***CXCL12A***	11	11	9	8	3	up
***CYP11B2***	6	5	3	2	3	down
***PRLR***	8	5	1	1	4	down

### qRT-PCR validation of gene expression

To validate the results obtained by RNA-seq, the 10 candidate genes with the highest k-core differences were selected from the co-expression network analysis for confirmation by qRT-PCR. Eight candidate genes (*SLC3A2*, *DPP4*, *CYP21A*, *CXCR3*, *CSF3R*, *CYP17A1*, *CXCL12A*, and *APOC1I*) showed k-core differences that were too low to allow proper quantification in both groups (sterile gonads and fertile gonads). The qRT-PCR analysis confirmed the outcome of the RNA-seq analysis regarding changes in expression of the 10 candidate genes (Table 6).

**Table 6:** Verification of gene expression changes by qRT-PCR.

Gene symbol	Expression in sterile gonads	Sequence of primers used in q-PCR	Expression of fold change (log2)	
			RNA-seq	qPCR
***TGFB3***	over	CAAGATTGGTCTGTCGGTA (F)	-1.25	-2.34
		AGCCAAACAGCGTTACAT (R)		
***CYP11A1***	under	ACGCCTGTTTGACCTCTG (F)	4.69	3.81
		TGAGCATTTCTGTTGGGAG (R)		
***CPA1***	over	GGTGTAAGCGTAGCCGTCAG (F)	-4	-3.85
		TCCAGGGAATGGGTGTCG (R)		
***CTSS***	over	CAAACAAGCGAGTTATGAT (F)	-1.36	-1.6
		AACCTCTTTCGGAGACAA (R)		
***BCL2L1***	under	GTGATGGACGAGGTGTTC (F)	1.22	0.19
		ATCCTGTCCACCAGCGAA (R)		
***ABCA1***	over	GCCACAGAGTGCCGTTAT (F)	-2	-3.1
		TTCCTTCGGGTGATGAGT (R)		
***TNFSF10***	over	CCTCGGTTCAAAGATGGAT (F)	-1.22	-2.18
		CGTTGGCAAAGCAAGGAA (R)		
***HSD3β***	under	TCGTATCCCGTCATCCAT (F)	1.84	0.73
		TGAGGTTTTCCTACAGCAAG (R)		
***CYP11B2***	under	TATTCTGCGTCCCAAACA (F)	4.67	3.78
		TTCGTCCAGAGCAGTATCA (R)		
***PRLR***	under	CAATAATGAGCCAATAACG (F)	5.12	4.06
		GTGGGTCTGTGGATGTTA (R)		

## Discussion

In general, the effects of inbreeding are first noticed in fertility related traits, especially in females [3]. However, in fish, only a few detailed studies have been performed on the fertility of DH individuals; these studies largely concentrated on delayed natural spawning time, decreased ovulation responses to hormonal induction, and reduced egg size and quality [12]. In this study, we used transcriptome sequencing to investigate the biological mechanisms of homozygotic sterility in female DH Japanese flounder.

Genes showing high levels of expression in fertile ovaries were primarily associated with steroid biosynthesis, while genes expressed highly in sterile gonads were mainly associated with other metabolic processes, such as immune response, cytokine–cytokine receptor interaction, and protein digestion and absorption. In the transcriptomes of sterile fish, seven genes showed the greatest upregulation and played important roles in steroid biosynthesis and in the Jak-stat signaling pathway: *CYP11A1*, *CYP11B2*, *CYP17*, *CYP21*, *HSD3B*, *BCL2L1*, and *PRLR*. Other researchers have confirmed that the variation in the expression of genes for steroid biosynthesis can affect hormone levels and cause metabolic disorders. For example, polycystic ovary syndrome, a common endocrine disease in women of reproductive age, is mainly caused by abnormal expression of steroidogenic enzymes, which also can induce abnormal follicular maturation [13]. *CYP11A1* is the first and rate-limiting step of steroid biosynthesis, and catalyzes the conversion of cholesterol to pregnenolone, the gene controls the rate of synthesis of all steroid hormones. Thus, *CYP11A1* is critical for steroidogenesis at the gonad development stage [10]. Additionally, *CYP11A1* is important for placental progesterone synthesis, which is essential for the maintenance of pregnancy in mammals [14]. In humans, mutation of *CYP11A1* can cause congenital lipoid adrenal hyperplasia, with a range of symptoms such as male sex reversal, high plasma adrenocorticotropic hormone levels, and increased plasma concentrations of gonadotrophins [15]. Similarly to humans, *Cyp11a1*-null mice have exceedingly high levels of adrenocorticotropic hormone but very little corticosterone and aldosterone in the plasma [16]. *CYP11A1* is also regarded as a critical regulator for gonadal development in fish. In zebrafish, *CYP11A1* is synthesized as a maternal transcript, and targeted knockdown of the gene leads to a shortened embryonic axis and epiboly defects, presumably because of the lack of adequate maternal steroid supply [17]. *CYP11A1* is also indispensable for gonadal development and maturation in catfish [18]. Here, *CYP11A1* was found to be expressed at extremely low levels in sterile ovaries, and at much higher levels in fertile ovaries. We suggest that *CYP11A1* has a significant effect on ovary development in the Japanese flounder and that lower levels of expression impair oogenesis.

*CYP11B2*, aldosterone synthase gene, encodes a cytochrome P450 membrane-bound heme-containing enzyme that accepts electrons from NADPH via accessory proteins; it participates in hydroxylation and other oxidative conversions of target molecules. *CYP11B2* is normally expressed only the in zona glomerulosa where it catalyzes three sequential reactions: 11β-hydroxylation, 18-hydroxylation, and 18-oxidation to form aldosterone [19–20]. Mutation of *CYP11B2* can cause aldosterone synthase deficiency in humans [21]. Fish *CYP11B* also catalyzes the 11β-hydroxylation step that produces 11β-hydroxytestosterone in the gonad [21]. Expression of *CYP11B* in fish shows a sexually dimorphic pattern in the gonad, as it only expressed in testis and not in the ovary of either developing or adult fish [19, 22–24]. In contrast, *CYP11B2* was expressed in DH ovaries with a higher level of expression in gonads of fertile compared to sterile flounder. We speculate that gonadal development in DH flounder may differ from the standard pattern.

Germ cell development in sterile DH ovaries was inhibited at an early stage and vitellogenesis was not completed to enable final oocyte maturation. It is known that sex steroids regulate liver metabolism to produce vitellogenin (VTG), the egg yolk precursor protein. Additionally, 17α,20β-dihydroxy-4-pregnen-3-one is an essential hormone for final oocyte maturation in fish [25–26] and *CYP17* has a vital role in its production in gonadal tissues and also for production of sex steroids [27]. The expression of *CYP17* in the ovaries of trout and eel increases during development and maturation of the follicles [28]. It was reported that the level of *CYP17* expression increases continually during gonad development in Japanese flounder [29]. Mutation of the coding region of *CYP17* or changes to its methylation status might influence expression and, consequently, reproductive endocrine levels [30]. In common carp, *CYP17* deficiency leads to inter-renal hyperplasia [31]. Our study also indicated that low expression of *CYP17* may affect the production of sex steroids and further restrict gonadal development.

In the transcriptome from sterile ovaries, the least expressed genes were *HSD3β* and *CYP21*. *HSD3β* is responsible for the second step of steroidogenesis, namely, the conversion of P5 into progesterone. *CYP21* catalyzes the final steps in the production of cortisol and functions in sex steroid production [32]. *CYP21* deficiency in humans leads to congenital adrenal hyperplasia, an autosomal recessive disorder associated with deficiency in adrenocortical enzymes necessary for cortisol biosynthesis [33]. Deficient expression of the *CYP21* gene results in the accumulation of 17-α-hydroxy progesterone and its conversion to androgens. Excessive adrenal androgen production can induce clinical hyperandrogenism, anovulatory cycles, and infertility [34]. In addition to the five genes described above, *PRLR* is also involved in steroid biosynthesis. *PRLR* is a member of the class 1 cytokine receptor superfamily and forms a transmembrane chain that is embedded in the cell membrane; it functions in combination with prolactin [35]. The role of *PRLR* in reproduction in mice was demonstrated through analysis of a germ-line null mutation. Female *PRLR*-/- mice exhibit total sterility because of the regression of the corpora lutea, the absence of sufficient progesterone to support implantation and the subsequent development and maintenance of the placenta [36]. *PRLR* mRNA and protein have been found in the gonads of some fish, such as Mozambique tilapia [37], Nile tilapia [38], and seabream [39]. Furthermore, PRLR levels often change during the breeding cycle. The highest PRLR level in female Nile tilapia plasma occurs after spawning and during vitellogenesis [40], suggesting that *PRLR* may be involved in vitellogenesis and/or ovulation. These observations are consistent with our experimental results that *PRLR* is expressed at low levels in sterile DH fish that show yolk accumulation deficiency. This implies that *PRLR* has an important role in ovarian development in Japanese flounder.

VTG is synthesized mainly in the liver under the regulation of E2. It is transferred to the oocyte via thecal capillaries to the granulosa layer, and passes to the oocyte surface through pore canals in the zona radiata. Subsequently, the VTG enters the cell by receptor-mediated endocytosis [41]. In the present study, we found upregulation of the *VTG* gene in sterile DH fish. *VTG* is involved in vitamin metabolic process, transcytosis, lipoprotein transport, proteolysis, and receptor-mediated endocytosis. All of these processes are related to yolk deposition (vitellogenesis). Thus, although the DH fish were sterile, they still showed higher expression of the gene for yolk protein accumulation. The numerous oocytes in the sterile gonad presumably still continue vitellogenesis. We speculate that steroid hormone disorder caused by key enzyme genes for steroid biosynthesis (as described above) is associated with the high level of *VTG* expression.

The vitellogenic period is characterized by high production of RNAs, proteins, lipids, vitamins, and hormones [42]. In the sterile ovaries, we found higher expression of genes related to cytokine–cytokine receptor interaction, and protein digestion and absorption, namely, *TGFB3*, *CSF3R*, *CXCR3*, *SLC3A2*, and *DPP4*. Of these, *TGFB3*, *CSF3R*, and *CXCR3* have been reported to be expressed in the preovulatory ovary of fish [43]. *SLC3A2* is expressed in a wide variety of tissues in mammals, specifically in the yolk syncytial layer in the embryo [44]. *DPP4* plays an important role in enzymatic degradation of incretin peptides [45]. We speculate that these genes are correlated to vitellogenesis in the sterile ovaries.

Follicular atresia is a widespread phenomenon in fish ovaries under both natural and experimental conditions; in this process, a number of ovarian follicles recruited into the vitellogenesis pool fail to complete maturation and ovulation [46]. Atresia mainly occurs during the post-spawning period, but can also be observed in other stages of the reproductive cycle [47]. In this study, although the appearance of the sterile DH gonads differed from that of follicular atresia in fish described in other studies [48–49], some genes related to atresia were more highly expressed in the sterile DH gonads. *APOC1-I* encodes a protein component of chylomicrons, high-density lipoproteins (HDLs) involved in lipid transport in the bloodstream [50]. *ABCA1* mediates the transport of cellular cholesterol and phospholipids to *APOA-1* to generate nascent HDL particles [51]. The expression of *ABCA1* and *APOC1-I* is required for the clearance of excess cholesterol and phospholipids from hepatocytes and for the reduction in hepatic lipid accumulation [52]. During follicular atresia in rainbow trout, there is massive transfer of the oocyte yolk proteins and possibly lipids into the bloodstream combined with HDLs [53] because of the ingestion and digestion of the yolk by the follicular cells. The study of Senegalese sole has shown the importance of lipid-metabolic process during follicular atresia in fish [54]. In humans, serum *APOC1-I* has been proposed to be an early marker for metabolic abnormalities in women with polycystic ovary syndrome [55]. Similarly, *APOC1-I* may be a useful marker to identify factors involved in premature ovarian regression in cultured fish [54]. Therefore, lipid-metabolic processes during follicular atresia in fish may have evolved to facilitate the redistribution of energy-rich yolk materials from oocytes that failed to develop properly [56]. In the sterile DH females, gonadal development arrested at the vitellogenic stage, presumably stimulating the redistribution of energy from the oocytes. The finding of high *APOC1-I* and *ABCA1* transcriptional levels in sterile ovaries of Japanese flounder provides additional evidence for this inference.

In the transcriptome of sterile ovaries, the genes showing the highest upregulation were related to immune response. During follicular atresia in fish, immune cells may act synergistically with follicular cells in the resorption of the oocyte through release of lytic enzymes [57]. Granulocytes appear in the atretic follicles of some fish species [54, 58], and there is evidence of a relationship between follicular regression and immune cells [48]. In this study, a series of chemokines (*CCR4*, *CMKLR1*, *CXCL12A*, and *CXCR3*) in the immune response process drew our attention. In the mammalian ovary, chemokines play an important role in follicular atresia and energy reassignment [57]. In atretic follicles of Senegalese sole, the chemokine *LECT2* shows high expression levels [54]. However, the molecular pathways through which chemokines act in atretic follicles are largely unknown. Ovarian expression of *CCR4*, *CMKLR1*, *CXCL12A*, and *CXCR3* have not yet been reported in fish and, therefore, the structural and functional relationships of these 4 chemokines genes in Japanese flounder requires further investigation. Atresia is considered as an apoptotic process in many organisms, and the proteolytic degradation of the oocyte yolk proteins, mediated by the differential activation of lysosomal cathepsins, has been proposed as the initial event leading to oocyte cell death [56]. Two key genes, *TNFSF10* and *CTSS*, involved in the regulation of cell apoptosis also appeared in the immune response process category. *TNFSF10* induces apoptotic cell death in cancer by binding to its functional death receptors [59]. *CTSS* is an important cathepsin that inhibits apoptosis; null mutation of the gene results in decreased DNA injury and apoptosis [60]. We speculate that non-functional oocytes in sterile DH gonads will become apoptotic and there will be a redistribution of cellular energy as a result of follicular atresia.

## Conclusions

In this study, we conducted a comprehensive comparison of gene expression in fertile and sterile gonads of DH Japanese flounder. We identified many genes responsible for gonad development through transcriptome sequencing. In combination with the sequencing results, we speculated that genes involved in steroid biosynthesis that showed reduced levels of expression might affect the development of the ovary and possibly cause sterility in DH Japanese flounder. We also found that genes related to apoptosis and to digestion of the yolk for energy reallocation showed higher expression in sterile DH gonads. These results indicated that the arrested oocytes might become apoptotic and that their energy might be redistributed. These significant changes in gene expression patterns provide further insights not only into DH reproductive dysfunction, but also into oogenesis in Japanese flounder and the molecular mechanisms of piscine oocyte maturation.

## Materials and Methods

### Ethics Statement

Experimental treatment of the Japanese flounder in this study was performed strictly in accordance with the Guide for Care and Use of Laboratory Animals of the Chinese Association for Laboratory Animal Sciences (No. 2011–2); all the experiments were approved by the animal care and use committee of Beidaihe Central Experiment Station.

### Screening of sterile DH Japanese flounder

The DH Japanese flounder were produced in April 2009, from a single wild female that captured from the Qinghuangdao area of The Bohai Sea and reared at Beidaihe Central Experiment Station, Qinghuangdao, following the standard protocol for mitogynogenesis [16].

From 2012, the DH females were manually checked for egg production every year. Eggs were extracted from fertile individuals, and the progenies survived. Approximately, 70% of DHs had a flat abdomen and appeared sterile. In May 2014, three sterile DHs that could not produce eggs were euthanized using 300 mg/L tricaine methanesulfonate (MS222), along with three fertile DHs who produced viable progeny. The gonads were dissected from each female, immediately frozen in liquid nitrogen, and stored at -80°C until use.

### RNA preparation, cDNA synthesis, sequencing, and de novo assembly

Total RNA for each sample was extracted with an RNeasy Mini Kit and digested with DNase I following the manufacturer’s instructions (Qiagen, Hilden, Germany). Integrity and size distribution of the RNA samples were verified using an Agilent 2200 Bioanalyser (Agilent Technologies, Germany). Samples with an RNA Integrity Number ≥ 8.0 were used for cDNA library preparation. The concentration of RNA in each extracted sample was measured using a Qubit 1.0 Fluorometer (Invitrogen, Carlsbad, USA). The cDNA libraries were constructed for each pooled RNA sample using the TruSeqTM RNA sample preparation kit (Illumina, Inc.) according to the manufacturer’s instructions. The tagged cDNA libraries were pooled in equal ratio and used for 101 bp paired-ends sequencing with an Illumina HiSeqTM 2000.

Clean reads were obtained from the raw reads by removing the adaptor sequences, reads with >5% ambiguous bases (noted as N), and low-quality reads containing more than 30 percent of bases with qualities of <20. Contig assembly was carried out using Trinity software [17] and the contig of the 6 different samples was clustered using CAP3 software [18] to achieve the final unigenes result. Gene Annotation was performed using tBlastx (National Center for Biotechnology Information; http://www.ncbi.nlm.nih.gov/) on zebrafish, mouse, and human transcripts and by filtering using the criterion “E-Value < 1e-5”. Coding sequence prediction was obtained using EST scan software [61]. BWA [62] software was used for mapping the reads to the unigene assembly and the counts were calculated with Picard software. We applied the DEseq algorithm to filter the differentially expressed unigenes, after the analyses of significance and false discovery rate (FDR), with the following criteria: i) Fold Change >2 or <0.5; and ii) FDR < 0.05 [63].

### Analysis of GO category, pathway, gene-act-network, and gene co-expression

Gene ontology (GO) analysis was performed to elucidate the biological implications of unique genes in the significant or representative profiles of differentially expressed genes in the experiment [64]. We downloaded the GO annotations from NCBI (http://www.ncbi.nlm.nih.gov/), UniProt (http://www.uniprot.org/), and Gene Ontology (http://www.geneontology.org/). Fisher’s exact test was applied to identify significant GO categories and FDR was used to correct the *p*-values. Pathway analysis was used to identify significant pathways associated with the differentially expressed genes according to the KEGG database. We also used Fisher’s exact test to select significant pathways, and the threshold of significance was defined by *p*-value and FDR [65]. The KEGG database was used to build a network of genes according to the relationships among genes, proteins, and compounds in the database. Gene Ontology is structured as a directed acyclic graph, and each term has defined relationships to one or more other terms. We built gene co-expression networks to show the relationships among genes [66]. Gene co-expression networks were based on normalized expression values of genes selected from those genes in significant GO terms and pathway terms. For each pair of genes, we calculated the Pearson correlation and chose the pairs with a significant correlation (FDR < 0.05) to construct the network [67]. In this network analysis, degree centrality was the most simple and important measure to determine the relative importance of a gene to the network. Degree centrality was defined as the number of links of one node to others [68]. The properties of the networks were also analyzed using k-cores, derived from graph theory as a method to simplify graph topology analysis. The k-core of a network is a sub-network in which all nodes are connected to at least k other genes. The k-core of a protein–protein interaction network usually contains cohesive groups of proteins [69].

### Real-time qPCR assays

Real-time qPCR was performed to validate gene expression data from the RNA-Seq analysis. Total RNA was reverse transcribed using a Superscript III kit (Invitrogen) according to the manufacturer’s instructions. Real-time qPCR was performed using a 7500 Fast Real Time PCR system (Applied Biosystems, Foster City, USA) with SYBR green I nucleic acid kit (Invitrogen) in 20 μL reactions. Each reaction consisted of 2 ng of total RNA and 0.5 μL of each primer. PCR was performed in a thermocycler under the following conditions: 2 min at 95°C, then 40 cycles of 10 s at 95°C, 30 s at 60°C, and 45 s at 72°C, followed by 5 min at 72°C. To verify the presence of a specific product, a melting curve analysis of amplification products was performed at the end of each PCR. Primer pairs for qPCR amplification were designed using Netprimer (http://www.premierbiosoft.com/netprimer/). The comparative CT method of quantification was used to quantify the relative expression of specific genes. All samples were run in triplicate, and the relative expression levels of target genes were calculated with the 2^-∆∆Ct^ method, with the β-actin gene used for normalization of the data.

## Supporting Information

S1 File Differential gene expression analysis. (XLS)

S2 File Go_analysis of the differential genes. (XLS)

S3 File Pathway_analysis of the differential genes. (XLS)
