# Correction: Gonadal Transcriptome Analysis in Sterile Double Haploid Japanese Flounder

**DOI:** 10.1371/journal.pone.0147668

**Published:** 2016-01-19

**Authors:** Xiaoyan Zhang, Jilun Hou, Guixing Wang, Hongbo Jiang, Yufen Wang, Zhaohui Sun, Xiufeng Jiang, Qinghai Yu, Haijin Liu

In the Funding section, the grant number from the funder National High Technology Research and Development Program in China is listed incorrectly. The correct grant number is: 2012AA10A408-05.
